# The histone deacetylase inhibitor SAHA acts in synergism with fenretinide and doxorubicin to control growth of rhabdoid tumor cells

**DOI:** 10.1186/1471-2407-13-286

**Published:** 2013-06-13

**Authors:** Kornelius Kerl, David Ries, Rebecca Unland, Christiane Borchert, Natalia Moreno, Martin Hasselblatt, Heribert Jürgens, Marcel Kool, Dennis Görlich, Maria Eveslage, Manfred Jung, Michael Meisterernst, Michael Frühwald

**Affiliations:** 1 Department of Pediatric Hematology and Oncology, University Childrens’ Hospital Muenster, Muenster, Germany; 2 Institute of Molecular Tumor Biology, WestfalianWilhelms University, Muenster, Germany; 3 Institute of Neuropathology, University Hospital Muenster, Muenster, Germany; 4 Division of Pediatric Neurooncology, German Cancer Research Center (DKFZ), Heidelberg, Germany; 5 Institute of Biostatistics and Clinical Research, WestfalianWilhelms University, Muenster, Germany; 6 Institute of Pharmaceutical Sciences, Freiburg, Germany; 7 Childrens’ Hospital Augsburg, Swabian Childrens’ Cancer Center, Klinikum Augsburg Stenglinstr 2, Augsburg 86156, Germany

## Abstract

**Background:**

Rhabdoid tumors are highly aggressive malignancies affecting infants and very young children. In many instances these tumors are resistant to conventional type chemotherapy necessitating alternative approaches.

**Methods:**

Proliferation assays (MTT), apoptosis (propidium iodide/annexin V) and cell cycle analysis (DAPI), RNA expression microarrays and western blots were used to identify synergism of the HDAC (histone deacetylase) inhibitor SAHA with fenretinide, tamoxifen and doxorubicin in rhabdoidtumor cell lines.

**Results:**

HDAC1 and HDAC2 are overexpressed in primary rhabdoid tumors and rhabdoid tumor cell lines. Targeting HDACs in rhabdoid tumors induces cell cycle arrest and apoptosis. On the other hand HDAC inhibition induces deregulated gene programs (*MYCC-, RB* program and the stem cell program) in rhabdoid tumors. These programs are in general associated with cell cycle progression. Targeting these activated pro-proliferative genes by combined approaches of HDAC-inhibitors plus fenretinide, which inhibits cyclinD1, exhibit strong synergistic effects on induction of apoptosis. Furthermore, HDAC inhibition sensitizes rhabdoid tumor cell lines to cell death induced by chemotherapy.

**Conclusion:**

Our data demonstrate that HDAC inhibitor treatment in combination with fenretinide or conventional chemotherapy is a promising tool for the treatment of chemoresistant rhabdoid tumors.

## Background

Altered states of chromatin in cancer cells are a promising novel target for therapeutic strategies in the treatment of malignant tumors. Two of many important mechanisms of epigenetic regulation are DNA methylation and histone acetylation, which are closely connected and deregulated in many malignancies [1,2]. HDAC inhibitors counteract cell proliferation and induce apoptosis by altering histone tails and non-histone targets including transcription factors, hormone receptors, signal transducers and molecular chaperones [3]. Recent investigations demonstrated that HDAC-inhibitors (HDACi) display selective toxicity against tumor cells and sensitize cancer cells to the cytotoxic effects of conventional cytostatic drugs [4-6]. These characteristics have led to the use of several HDACi in a number of single agent or combinatorial clinical trials (more than 100 currently listed) (e.g. in lung, breast bladder cancer, glioblastoma, leukemias and lymphomas) [7,8]. Recently the importance of deregulation of epigenetic mechanisms in the development of embryonal tumors such as medulloblastoma, CNS PNET and AT/RT has been demonstrated. Epigenetically active compounds including histone deacetylase inhibitors (HDACi) and demethylating agents (e.g. azacitidine) have been identified as attractive tools for the treatment of embryonal tumors, including rhabdoid tumors [9-11].

Rhabdoid tumors are rare but highly aggressive neoplasms with an incidence peaking between birth and 3 years of age [12]. Rhabdoid tumors of the brain are termed atypical teratoid/rhabdoid tumors (AT/RT), however rhabdoid tumors can also be found in soft tissues (MRT, malignant rhabdoid tumors) and the kidneys (RTK, rhabdoid tumor kidney). Outcome especially for the youngest patients with rhabdoid tumors remains bleak despite the use of aggressive multimodal chemotherapeutic, radiotherapeutic and surgical interventions (2-year survival rates between 15% to 55% for children with AT/RT) [13,14]. The majority of rhabdoid tumors exhibit biallelic alterations in the tumor suppressor gene *SMARCB1*. Apart from *SMARCB1* mutations only very few and rather infrequent further alterations have been detected [15,16]. Some pathways drivingoncogenesis are defined in rhabdoid tumors: In *SMARCB1* negative tumors oncogenes (including *MYC* and *CYCLIND1*) [17-20] and tumor cascades such as the sonic hedgehog pathway are activated [19]. Furthermore, *SMARCB1* acts as a direct repressor of the polycomb complex subunit EZH2 [21]. SMARCB1 and EZH2 exhibit antagonistic functions in the regulation of stem cell-associated programs. In rhabdoid tumors loss of *SMARCB1* activates those programs [21].

Here we demonstrate that several HDACs, including HDAC1 and 2, are overexpressed in primary rhabdoid tumors and tumor cell lines. The histone deacetylase inhibitor (HDACi) SAHA inhibits cell proliferation of rhabdoid tumor cells by inducing a reversible G_2_-arrest and subsequently apoptosis. Interestingly SAHA activates tumor pathways, which are already deregulated in rhabdoid tumors (such as *MYC, CYCLIND* and the pluripotency associated program controlled by *EZH2*). Based on these results we developed a targeting strategy combining SAHA with fenretinide, which suppresses cyclinD1, and SAHA with conventional chemotherapy. These combinations showed strong synergistic effects on tumor cell growth and represent a promising potential tool for the treatment of rhabdoid tumors.

## Methods

### Cell lines

Rhabdoid tumor cell lines BT12 and BT16 (AT/RT), G401 (rhabdoid tumor of the kidney (RTK)) and A204 (rhabdoid tumor of the liver) were cultured in DMEM high glucose formulation (Invitrogen, Karlsruhe, Germany), supplemented with 10% fetal bovine serum (South American, Invitrogen), 2% glutamine (Invitrogen, Karlsruhe, Germany) and no additional antibiotics. The cells were cultured at 37°C in a humidified atmosphere with 5% CO_2_. A204 and G401 were obtained from ATCC. BT12 and BT16 were a gift from Dr. P. Houghton. Mouse embryonic stem cell (ESC) line OG_2_ was cultured to the distributors recommendation in DMEM with Glutamax, non-essential aminoacids, mercaptoethanol, PenStrep (all PAA Laboratories, Pasching, Austria) and LIF. For differentiation of ESCs OG_2_ cells were cultured at least five days without LIF. OG_2_ cell line was a gift from Hans Schöler (MPI Muenster, Germany).

The identity of all cell lines was verified using ST-PCR. All experiments using cell lines in this publication were at least performed using three independent replicates.

### Histone deacetylase inhibitors, Cyclin D inhibitors and chemotherapy

Suberoylanilindehydroxamic acid (SAHA) (Merck, Darmstadt, Germany), Trichostatin A (TSA) (Sigma, Taufkirchen, Germany), N-(4-hydroxyphenyl)retinamide (4-HPR or fenritinide) (ONBIO, Ontario, Canada, # 65646-68-6) and 4-Hydroxy-Tamoxifen (4OH-Tam) (Sigma Taufkirchen, Germany, # H7904) were reconstituted in 100% ethanol, as a 10 mM solutions. M344 was synthesized by one of us (M.J.). Doxorubicin was purchased from Merck (Merck Millipore, Darmstadt, Germany # 324380).

### Cytotoxicity assay

Cell suspensions (5,000 cells/100 μl) were seeded into four 96-well-plates. Cells were allowed to reach exponential growth before 100 μl of cell culture medium containing the drugs at different concentrations were added. Each drug concentration (0, 0.01, 0.1, 1, 10 and 100 μM) was tested in 3 biological replicates. For experiments with combined treatment we used compound 1 (see Tables 1 and 2) in increasing concentrations as in single compound experiments (0, 0.01, 0.1, 1, 10 and 100 μM). Compound 2 was used at 1/10 of the concentration of compound 1. After 0, 24, 48 and 72 hr cells were incubated 3 hr with 10 μl MTT reagent (5 mg/ml MTT dissolved in PBS). Metabolically active cells cleaved the yellow tetrazolium salt to a purple formazan dye. A decrease in the number of living cells correlated with the number of purple formazan crystals. Crystals were dissolved in 100μllysis buffer. The specimen was evaluated spectrophotometrically at 570 nm and a reference of 650 nm using a Multiskan Ascent multiplate reader (Labsystems, Helsinki, Finland).

**Table 1:** Summarizes results of MTT-tests in different rhabdoid tumor cell lines (A204, G401, BT16) treated with HDAC-inhibitors (SAHA, TSA, M344) cyclin D inhibitors (fenretinide, tamoxifen) as single compounds and in combinations of both classes of compounds

**Cell line**	**Compound 1**	**Compound 2**	**IC 50 μM**	**m**	**CI**	**R**^**2**^
**A204**	SAHA	---------------	24.72	0.6	---------------	0.72
**A204**	M344	---------------	128.76	0.57	---------------	0.67
**A204**	TSA	---------------	1.83	0.43	---------------	0.87
**A204**	Tam	---------------	2.67	0.5	---------------	0.86
**A204**	Fen	---------------	1.87	0.4	---------------	0.84
**A204**	SAHA	Tam	0.97	0.36	0.07	0.75
**A204**	SAHA	Fen	1.25	0.48	0.1	0.72
**A204**	M344	Tam	0.97	0.48	0.19	0.56
**A204**	M344	Fen	0.28	0.24	0.01	0.88
**A204**	TSA	Tam	0.16	0.2	0.08	0.77
**A204**	TSA	Fen	0.1	0.24	0.05	0.73
**G401**	SAHA	---------------	31.82	0.44	---------------	0.87
**G401**	Tam	---------------	3.13	0.53	---------------	0.89
**G401**	Fen	---------------	3.37	0.54	---------------	0.85
**G401**	SAHA	Tam	1.42	0.3	0.06	0.9
**G401**	SAHA	Fen	1.65	0.54	0.09	0.91
**BT16**	SAHA	---------------	8.39	0.64	---------------	0.93
**BT16**	Tam	---------------	2.09	0.75	---------------	0.9
**BT16**	Fen	---------------	2.74	0.5	---------------	0.91
**BT16**	SAHA	Tam	0.11	0.44	0.02	0.87
**BT16**	SAHA	Fen	0.43	0.52	0.06	0.86

Table shows results after 72 h of treatment.

CI = combination index [22].

**Table 2:** Summarizes results of MTT-tests in different rhabdoid tumor cell lines (A204, G401, BT16) treated with HDAC-inhibitors (SAHA, TSA, M344) or doxorubicin as single compounds or in combinations of both compounds

**Cell line**	**Compound 1**	**Compound 2**	**IC 50 μM**	**m**	**CI**	**R**^**2**^
**A204**	SAHA	---------------	24.72	0.6	---------------	0.72
**A204**	DOXO	---------------	6.48	0.37	---------------	0.72
**A204**	DOXO	SAHA	0.16	0.22	0.02	0.76
**G401**	SAHA	---------------	31.82	0.44	---------------	0.87
**G401**	DOXO	---------------	0.67	0.38	---------------	0.77
**G401**	DOXO	SAHA	0.03	0.17	0.03	0.85
**BT16**	SAHA	---------------	8.39	0.64	---------------	0.93
**BT16**	DOXO	---------------	0.13	0.18	---------------	0.83
**BT16**	DOXO	SAHA	0.003	0.2	0.02	0.81

The CI values have been determined at the respective IC50 concentration. CI < 1 indicates synergism. R^2^ denotes the coefficient of determination of the linear regression in the median effect plot.

### Analysis of combined drug effects on cytotoxicity

To evaluate drug combination effects we analyzed cytotoxicity assay data using the median effect method by Chou and Talalay [22]. We employed three biological replicates of the cytotoxicity assay for each experiment. The fraction of unaffected cells was defined as the proportion of living cells compared to the control. The combination index indicates synergism if CI < 1, antagonism for CI > 1 and an additive effect for CI = 1. Values of the CI were determined at the IC_50_ concentration (fraction affected = 0.5). The method was implemented in the statistical software R (Version 2.15.1).

### Western blots

For differentiation of mouse embryonic stem cell line OG_2_ cells were grown without LIF. After 5d cells were harvested and lysed using Biorupture (Diagenode; Liege, Belgium). SDS page was performed as described [9]. Briefly tris/glycine gels were used for 1-D separation (20 mg protein per lane). Semidry transfer was carried out for 1 h at 18 V using tris/glycine buffer [9]. Western-blots were scanned and aligned with the Photoshop 6.0 channel mixer (Adobe).

### Antibodies for western blots

Hdac1 (ab7028) rabbit polyclonal 65 kDA, 1:500, (Abcam, Cambridge UK)

Hdac2 (ab12169) mouse monoclonal, 56 kDA, 1:500, (Abcam, Cambridge UK)

α-Tubulin (sc 23948) mouse monoclonal, 50–55 kDa, 1:1000, (Santa Cruz, Heidelberg, Germany)

Oct4 (sc-8628) goat polyclonal, 43–50 kDa, 1:500, (Santa Cruz, Heidelberg, Germany)

CyclinD1 (sc 754), rabbit polyclonal, 38 kDa, 1:500, (Santa Cruz, Heidelberg, Germany)

H3K27me3 (6002), mouse monoclonal, 18 kDa, 1:500, (Abcam, Cambridge UK)

Ezh2 (AC22), mouse monoclonal, 98 kDa, 1:500, (Cell Signaling, Danvers, USA)

### Apoptosis detection and cell cycle analysis

Effects on apoptosis induction were analyzed in A204 cells. Cells were incubated in 75 cm^2^ tissue flasks with the drugs for 24, 48 and 72 hr. A204 cells were treated with ethanol (control), with SAHA (1 μM or 10 μM), fenretinide (1 μM or 10 μM) or a combination of SAHA (1 μ or 10 μM) and fenretinide (1 μM or 10 μM). All experiments were at least performed in biological triplicates. An annexin-V-FITC apoptosis detection kit was employed (BD Biosciences, Heidelberg, Germany). Cells were washed with PBS and fluorescein isothiocyanate-conjugated annexin-V and propidiumiodide were added. Cells were then incubated at room temperature (15 min) and analyzed by flowcytometry, using a Facscalibur (BD Biosciences, Heidelberg, Germany). For cell cycle analysis cells were cultured and treated with compounds as described before, incubated with DAPI and measured using the Facscalibur(BD Biosciences, Heidelberg, Germany).

### cDNA microarray experiments and statistical analysis

A204 cells were treated with 10 μmol SAHA or equal amounts of ethanol (control). SAHA treated A204 cells and control samples were used as biological triplicates. After 12 h incubation cells were harvested and RNA was isolated by using an RNAeasy mini kit (Qiagen, Hilden, Germany). Affymetrix Gene Chip human 1.0 was used. Microarray data were analyzed using GeneSpring GX Software (Agilent, Santa Clara, USA). Microarray data complywiththe MIAME standard. Data were corrected for background noise, normalized and summarized using ExonRMA16 Algorithm. Following quality control was performed.

To identify differentially expressed genes in SAHA treated compared to untreated A204 cells we used an unpaired *t*-test. For further analysis we considered genes with a students *t*-test p-value of < 0.05 and a foldchange of ≥ 2. Prior published microarray data were used as supplied, as processed lists or downloaded from GEO [23,24]. Analysis of enriched GeneSets with GSEA (http://www.broadinstitute.org/gsea/index.jsp). GeneSets were downloaded from the MSig database [23,24]. To process the data, in-house scripts were employed.

For analysis of HDAC RNA expression we compared available data from geo database of primary rhabdoid tumors [25] to expression data from normal brain tissue [26]. These data were MAS5.0 normalized. HDACs in primary rhabdoid tumor were compared to normal brain tissue from different localizations of the brain.

Microarray data were confirmed using real-time qPCR (Step One plus, Applied Biosystem, Carlsbald, USA). RNA was isolated as described above from G401 cell treated with SAHA for 12 h. RT-PCR was performed using Takara RT-PCR kit (Clontec Laboratories, Mountain View, USA) according to the manufacturer’s protocol. For Real-time PCR we used Fast SYBR green (Applied Biosystem, Carlsbad, USA).

### Primers used for real-time PCR

hHMGB2 for: CGG-GGC-AAA-ATG-TCC-TCG-TA

hHMGB2rev: CGG-AAG-AGT-CCG-GGT-GTT-T

hBLM for: CAG-ACT-CCG-AAG-GAA-GTT-GTA-TG

hBLM rev: TTT-GGG-GTG-GTG-TAA-CAA-ATG-AT

hRFC3 for: GTG-GAC-AAG-TAT-CGG-CCC-TG

hRFC3 rev: TGA-TGG-TCC-GTA-CAC-TAA-CAG-AT

hMELK for: TCT-CCC-AGT-AGC-ATT-CTG-CTT

hMELK rev: TGA-TCC-AGG-GAT-GGT-TCA-ATA-GA

hMCM4 for: GAC-GTA-GAG-GCG-AGG-ATT-CC

hMCM4 rev: GCT-GGG-AGT-GCC-GTA-TGT-C

hMCM7 for: CCT-ACC-AGC-CGA-TCC-AGT-CT

hMCM7 rev: CCT-CCT-GAG-CGG-TTG-GTT-T

hPOLD3 for: GAG-TTC-GTC-ACG-GAC-CAA-AAC

hPOLD3 rev: GCC-AGA-CAC-CAA-GTA-GGT-AAC

## Results

### HDACs are highly expressed in primary rhabdoid tumors and rhabdoid tumor cell lines

**Figure 1:**
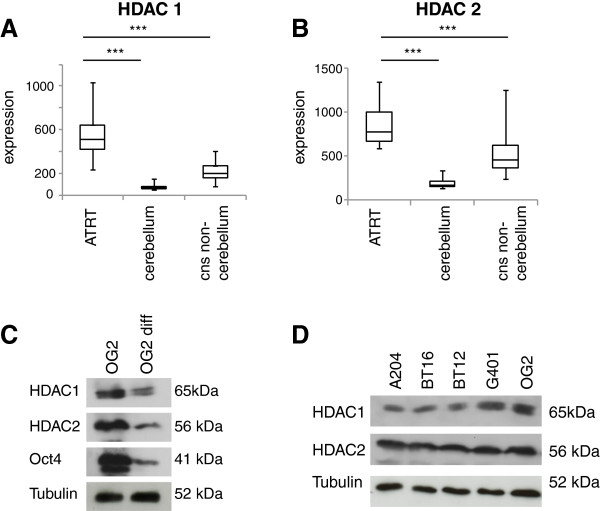
**Expression of HDACs in rhabdoid tumors. A** and **B**. HDACs are highly expressed on RNA level in primary rhabdoid tumors (n = 23) in comparison to differentiated brain tissue (n = 169) using available gene expression profiles of AT/RT [24] and different normal brain tissues [26]. **C**. HDAC1 and HDAC2 are highly expressed in mouse embryonic stem cells (ESC cell line OG_2_) and are down regulated after five days of differentiation (without LIF). **D**. Western-Blots of *SMARCB1* negative rhabdoid tumor cell lines (BT12, BT16, A204, G401) show high expression of HDAC 1 and HDAC 2, which is comparable to the expression of these HDACs in embryonal stem cells (OG_2_).

Aberrant expression of different HDACs has been observed in various tumors [1,2,9] and has been linked to tumor growth progression and poor outcome [27]. To compare the expression of HDACs in primary rhabdoid tumors and normal brain tissue we analyzed RNA expression profiles of AT/RT tissue [25] and normal brain tissue (Figure 1A and B and Additional file 1: Figure S1) [26] from datasets available in the GEO database [25,26]. Several HDAC including HDAC1, 2, 5, 6, 9 and SIRT1 are highly expressed in primary AT/RT (Figure 1A and B, Additional file 1: Figure S1).

Group 1 HDACs (including HDAC1, 2 and 3) are highly expressed in embryonic stem cells (ESCs) and down regulated during differentiation (Figure 1C) [28]. Comparing protein expression in different *SMARCB1* negative rhabdoid tumor cell lines (A204, G401, BT16, BT12) with ESCs (OG_2_; as a control with known highly expressed HDAC1 and HDAC2) demonstrate that group 1 HDAC levels are similarly expressed in rhabdoid tumors and ESC (Figure 1D).

Overall these data demonstrate that several HDAC are highly expressed in *SMARCB1* negative primary tumors and tumor cell lines.

### The non-selective histone deacetylase inhibitor SAHA induces reversible G_2_-arrest and apoptosis in *SMARCB1* negative tumors

**Figure 2:**
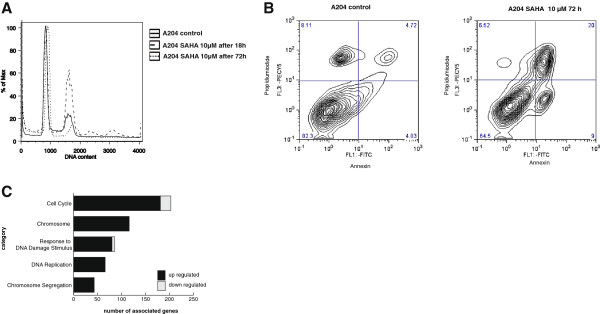
**Functional effects of SAHA in rhabdoid tumor cells. A**. Flow cytometry analysis: After 18 h treatment, SAHA (10 μM) induces G2 arrest and the formation of multinuclear cells (dashed line) after 18 h treatment in A204. After 72 h this G2 arrest is reversed (dotted line). **B**. SAHA (10 μM) treatment results in induction of apoptosis after 72 h. **C.** Gene ontology of RNA Microarrays show that many genes involved in "cell cycle", "DNA damage" and "chromosome segregation" are affected due to SAHA treatment

To evaluate whether high expression levels of HDACs correlate with cell cycle progression in rhabdoid cells we inhibited HDACs using the non-selective HDAC inhibitor (HDACi) SAHA (suberoylanilindehydroxamic acid) [9]. HDACi cause strong inhibition of cell growth in high-risk embryonal tumors of the central nervous system, including rhabdoid tumors [9,29]. Here we demonstrate that SAHA transiently (after 18 h) induces G_2_ arrest (Figure 2B, dashed, green line and Table 3). In contrast to published data demonstrating that the G_2_ arrest due to HDACi maybe a sign of resistance of cell lines to HDACi [30], rhabdoid tumor cell lines overcome the G_2_ arrest after 72 h (Figure 2B, dotted, blue line). After overcoming G_2_ arrest (Figure 2A and Additional file 2: Figure S2a) apoptosis is induced (Figure 2B and Additional file 2: Figure S2b).

**Table 3:** Shows %-values of G1-, S-, G2-phase cells of two different rhabdoid tumor cell lines (A204, G401) treated with 10 μM SAHA for 18 h or 72 h

**Cell line**	**G1-phase %**	**S-phase %**	**G2-phase %**
**A204 control**	57.0 +/− 1.2	21.1 +/− 0.9	22.0 +/− 2.3
**A204 SAHA 18 h**	43.3 +/− 2.1	10.5 +/− 0.6	46.3 +/− 3.4
**A204 SAHA 72 h**	79.1 +/− 1.9	5.3 +/− 0.4	15.6 +/− 0.9
**G401 control**	45.8 +/− 1.0	39.2 +/− 1.6	14.9 +/− 0.9
**G401 SAHA 18 h**	56.4 +/− 7.6	12.8 +/− 0.2	30.8 +/− 2.6
**G401 SAHA 72 h**	76.2 +/− 5.5	10.3 +/− 2.8	13.5 +/− 0.6

### SAHA induces expression of *RB-, MYC-* and pluripotency-associated genes

One major goal of our investigation was to identify potential combinatorial approaches of SAHA with other compounds based on molecular *in vitro* findings.

**Figure 3:**
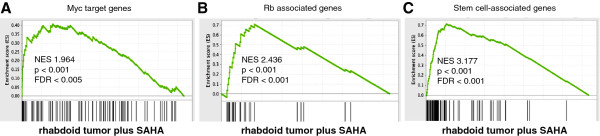
**SAHA induces pro-proliferative programs. A–C**. Microarrays were performed after treatment of rhabdoid tumor cell line A204 for 12 h with HDAC inhibitor SAHA. Gene set enrichment analysis (GSEA) [23,24] demonstrate that gene sets of *MYC* (**A**), Rb associated (**B**) and stem cell associated (**C**) are positively enriched in SAHA-treated rhabdoid tumor cell line A204. Genes on the X-axis show the overlap between the defined gene set and the regulated genes in the experiment. NES- negative enrichment score; FDR- false discovery rate (for brief description of statistics see http://www.broadinstitute.org/gsea/doc/GSEAUserGuideFrame.html).

To analyze known deregulated pathways in rhabdoid tumors, like RB and MYC, we performed microarray analysis of A204 after treatment with HDAC inhibitor SAHA. With a threshold of a 2-fold change we detected 1125 genes downregulated and approximately the same number of genes upregulated (1.119 genes). We analyzed known deregulated pathways in rhabdoid tumors, like cdk4/6-cyclinD-*RB*- and *MYC*, using gene set enrichment analysis (GSEA). We expected due to the observed growth arrest that these pro-proliferative pathways were downregulated after HDACi treatment [31]. Surprisingly these gene sets (*MYC*, *RB*, stem cell programs; Figures 3A-C) were not downregulated, but instead even more pronounced and highly significantly enriched following SAHA application. In these gene sets we demonstrated that target genes of MYC (Figure 3A), the RB-pathway (Figure 3B and Additional file 3: Figure S3) and genes associated with pluripotency (Figure 3C) are upregulated in SAHA-treated cells, indicating that not only apoptosis but also pro-proliferative pathways are induced by SAHA. Microarray data were validated in A204 and G401 rhabdoid tumor cell lines using qPCR (Additional file 3: Figure S3).

### SAHA synergizes with fenretinide in inhibiting rhabdoid cell growth

Treatment of rhabdoid tumor cell line A204 with SAHA upregulates RB- and MYC- target genes and the pluripotency-associated program controlled by EZH2. These genes and gene pathways induce pro-proliferative signals in rhabdoid tumors [21,32]. Based on these results we developed a combined targeting strategy. We tested treatment of SAHA in combination with tamoxifen and fenretinide. Both compounds affect the transcription as well as the protein stability of cyclin D1 [33,34]. Furthermore we combined SAHA with conventional chemotherapy (doxorubcin).

**Figure 4:**
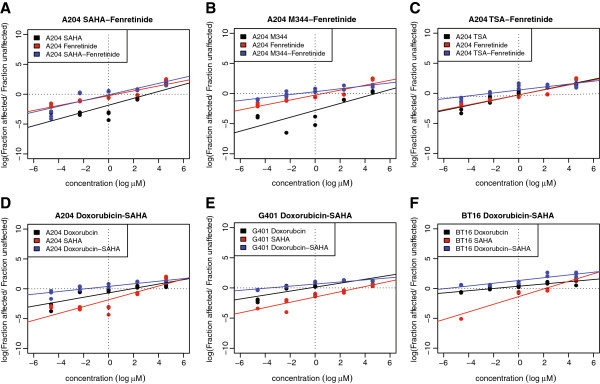
**Synergistic growth inhibition using SAHA with fenretinide and with conventional chemotherapy in rhabdoid tumor cell lines. A**, **B**, **C**. HDACi (SAHA, M344, TSA) were used in concentrations ranging from 0.01 μM to 100 μM. In single compound experiments fenretinide was used in the same increasing concentration (0.01 μM to 100 μM). In the combined approach we used HDACi (SAHA, M344, TSA) from 0.01 μM to 100 μM in combination with 10% fenretinide (0.001 μM to 10 μ). Median effect plots show that, SAHA and other HDACi (M344 and TSA) act strongly synergistic with the cyclinD inhibitor fenretinide (for CI-values see also table 1). **D**, **E**, **F**. Three different rhabdoid tumor cells lines (A204, G401, BT16) were treated with SAHA, doxorubicin or combinations of both compounds for 72 h and were analysed using MTT-assays. Median effect blots demonstrate that conventional chemotherapy (doxorubicin) acts synergistically with SAHA on inhibiting cell proliferation.

The Rb-pathway is controlled by phosphorylation of Rb by cdk4/6/cyclin D1. Dragnev*et al* showed that targeting cyclin D1 by fenretinide leads to G_0_-arrest and apoptosis in rhabdoid cell lines [34]. We compared cell proliferation effects of SAHA in rhabdoid cell lines as a single compound and combined treatment using SAHA with drugs that inhibit cyclinD1 (fenretinide and tamoxifen). The combination of these two groups of compounds demonstrated strong synergistic effects resulting in a significant decrease of the IC_50_ values compared to the IC_50_ of HDACi alone (Figure 4A-C and Table 1). The combination of 4-Hydroxytamoxifen (4-OH-Tam) and HDACi showed strong synergism, however the combination of fenretinide with HDACi reduces the IC_50_ values of the HDACi to a nanomolar range. Different HDAC inhibitors (SAHA, TSA, M344) in combination with fenretinide or tamoxifen in different rhabdoid tumor cell lines (Figure 4A-C and Table 1) showed strong synergistic effects. Using high concentrations of these inhibitors no synergism is observed due to cell toxicity of each single compound.

We additionally tested a treatment strategy combining doxorubicin with SAHA. This resulted in a clear reduction of doxorubicin IC_50_ values (Figure 4E and F; Table 2).

**Figure 5:**
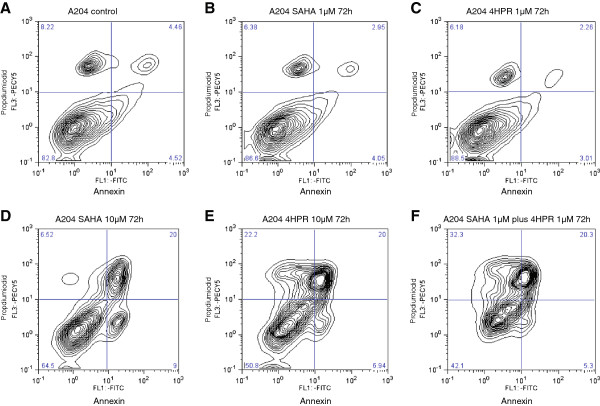
**HDACi and fenretinide act synergistic on induction of apoptosis.** A204 cells were treated for 72 h with HDACi SAHA (1 μM, 10 μM), fenretinide (4HPR) (1 μM, 10 μM) or combinations of both compounds. Low concentrations (1 μM) of SAHA (**B**) or fenretinide (**C**) as single treatment do not induce apoptosis compared to control (**A**). High concentrations (10 μM) of SAHA (**D**) or high concentrations of fenretinide (**E**), as well as low concentrations of combined treatment of SAHA plus fenretinide, induce apoptosis (**F**).

Using apoptosis assays we demonstrated, that the combination of SAHA and cyclinD1 inhibitors acts synergistically due to induction of apoptosis (Figure 5A-F and Table 4).

**Table 4:** S**hows percentage of rhabdoid tumor cell lines (A204, G401) surviving, in early or in late apoptosis after 72 h of treatment with SAHA as a single compound or in combination with 4HPR**

**Cell line**	**Control**	**SAHA 1 μM**	**SAHA 10 μM**	**4HPR 1 μM**	**4HPR 10 μM**	**SAHA 1 μM**
						**4HPR 1 μM**
**A204**						
**% surviving cells**	85.1 +/− 2.6	87.5 +/− 0.2	66.7 +/− 0.6	87.8 +/− 1.4	49.1 +/− 1.1	40.2 +/− 0.8
**% early apoptosis**	4.8 +/− 0.1	4.1 +/− 0.2	8.5 +/− 0.2	6.2 +/− 1.0	7.7 +/− 0.5	6.9 +/− 0.4
**% late apoptosis**	10.1 +/− 2.5	8.4 +/− 0.3	24.8 +/− 1.5	8.3 +/− 0.7	43.1 +/− 0.7	6.9 +/− 0.4
**G401**						
**% surviving cells**	90.3 +/− 0.8	91.2 +/− 1.5	64.7 +/− 2.9	92.3 +/− 2.2	60.0 +/− 2.2	62.9 +/− 3.2
**% early apoptosis**	5.2 +/− 0.6	5.1 +/− 0.9	23.6 +/− 0.9	4.1 +/− 1.2	26.9 +/− 0.7	27.3 +/− 1.3
**% late apoptosis**	4.5 +/− 0.2	3.8 +/− 0.7	11.7 +/− 2.1	3.6 +/− 1.0	13.1 +/− 1.4	9.8 +/− 4.2

## Discussion

Conventional chemotherapeutics remain disappointing in the treatment of rhabdoid tumors [35], making alternative approaches highly needed. Rhabdoid tumors seem to lack other mutations than those found in *SMARCB1*[15,36], suggesting epigenetic changes high likely in this tumor entity [15,37].

One of the most promising epigenetic targets for therapy of rhabdoid tumors is the inhibition of histone deacetylases by small compounds (histone deacetylase inhibitors (HDACi)) [9,11,38]. The rationale to use HDACi in rhabdoid tumors is simple. First, several HDACs (including HDAC 1, 2, 5, 6, 9 and SIRT1) are, like in many other tumor entities [1,2], overexpressed in rhabdoid tumors. Second, unselective HDACi inhibit cell growth, induce apoptosis and autophagy in rhabdoid tumor cell lines [9,38,39]. Third, HDACi lead to increased acetylation of histones making chromatin more accessible to transcription factors. SMARCB1, one of the core subunits of the SWI/SNF complex, is involved in ATP-dependent chromatin remodeling and modulation of accessibility of chromatin to transcription factors. As HDAC inhibition has been shown to restore imprinted tumor suppressors such as CDKN1C in rhabdoid tumors [39], we hypothesized that HDACi might generally compensate the missing chromatin remodeling function caused by *SMARCB1* loss. We investigated if HDAC inhibition leads to general restoration of known deregulated pathways in rhabdoid tumor cell lines (like *MYC-* or *RB-*pathways). Gene set enrichment analysis (GSEA) demonstrated that gene programs, which are deregulated by loss of *SMARCB1* in rhabdoid tumors (MYC, cyclin D1 and the pluripotency program) are further upregulatedfollowing SAHA treatment. These results suggest that HDAC inhibitors not only restore imprinted tumor suppressor genes, like *CDKN1C*[39], but also, as an “unselective transcription activator” increase expression of deregulated oncogenes in rhabdoid tumors. Based on these results we developed a combined targeting strategy using SAHA with conventional chemotherapeutics and compounds affecting cyclin D1-expression. The cdk4/cdk6/cyclin D1 pathway is directly controlled by *SMARCB1*[17,20,32]. Cyclin D1 forms a complex with cdk4/cdk6, which than phosphorylates Rb, thereby activates E2F1 and promotes cell cycle progression [40].

Combined targeted therapy of rhabdoid tumors makes sense from a molecular biology and from a clinical point of view. In other tumor entities including a subset of medulloblastomas individual pathways such as the sonic hedgehog pathway (SHH) seem to drive tumorigenesis [41]. This type of medulloblastoma has been shown *in vivo* to be highly responsive to small molecular compounds specifically inhibiting the sonic hedgehog pathway [42].

**Figure 6:**
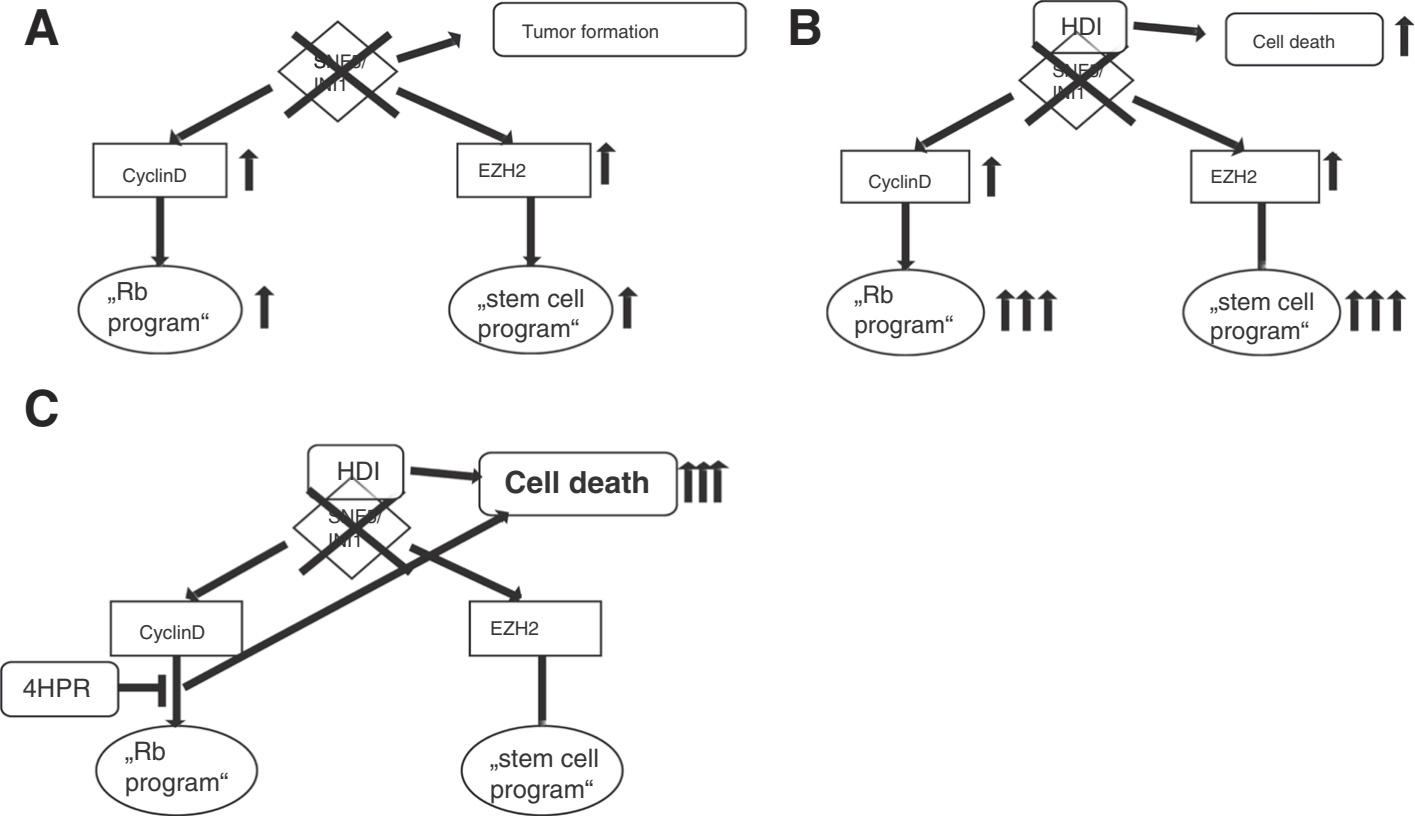
**Model of synergism of HDACi and fenretinide in rhabdoid tumors. A**. Loss of INI1 in rhabdoid tumors lead to tumor formation by deregulating different tumor pathways like cyclin D-Rb-pathway and “EZH2-stem cell program”. **B**. HDAC inhibition in rhabdoid tumor cell lines induces apoptotic cell death. On the other hand HDAC inhibition induces genes and pathways which are known to be already deregulated in this tumor entity (like cyclinD1 and “stem cell program”). 6. HDAC inhibition induces Rb-program by induction of CDK4/6/cyclin D1. Blocking HDAC mediated cyclin D induction by fenretinide results in dramatic induction of apoptosis. The combined inhibition of HDACs and cyclin D synergizes in the induction of apoptosis.

In rhabdoid tumors the situation might be somewhat different as biallelic mutation of the chromatin remodeling factor *SMARCB1* deregulates multiple tumor pathways (SHH, polycomb mediated pathways and Rb mediated pathways) (Figure 6). As we have demonstrated inhibition of one deregulated process (e.g. HDAC inhibition) may fail to target other deregulated cascades or even upregulate those pathways (like cdk4/6/cyclin D) due to an “unselective” transcriptional activation induced by HDACi. The current knowledge of the function of molecular pathways, the clinical behavior of rhabdoid tumors and our presented results make combined targeted therapy highly attractive and necessary for rhabdoid tumors. Inhibition of cyclinD1 and HDAC seems to affect two different deregulated targets in rhabdoid tumors, act synergistically and might be an attractive therapeutic approach for rhabdoid tumor treatment.

HDAC inhibitors as well as fenretinide have been evaluated in recent clinical phase I/II studies.

The bioavailability of fenretinide in children has been discussed controversially. In a recent study in pediatric neuroblastoma patients on fenretinide showed low bioavailability [43]. New formulations of fenretinide are presently evaluated [43].

Currently, over 100 phase I/II clinical trials are underway evaluating the safety and efficacy of HDAC inhibitors [44,45]. Clinical approaches with single use of HDACi show side effects like myelosuppression, fatigue and other toxicity and demonstrate only moderate effects on tumor growth of most tumor entities tested so far [45].

SAHA has been the first HDACi approved by the FDA and has been tested in several clinical trials. In clinical studies the effect of single use of HDACi seems to be minor, so combined strategies of SAHA with other compounds are tested [29]. In adult AML patients phase II studies showed that combined treatment of vorinostat (SAHA) with idarubicine and cytarabine is safe [46]. Other phase I/II studies demonstrated the safety of SAHA in combinations with paclitaxel and bevacizumab [47], with gemtuzumab [48] and bortezomib [49]. Vorinostat in pediatric patient cohorts has been well tolerated [50].

## Conclusion

To summarize our results we have demonstrated that

1. HDACi not only restore tumor suppressor genes like *CDKN1C*, but also induce pro-proliferative genes like *CyclinD1*, *MYC* and pluripotency associated genes

2. therapy of HDACi with cyclinD1 inhibitors and combined use of HDACiwith conventional chemotherapy demonstrates strong synergism on inhibition of tumor cell growth.

These experiments provide the rationale for a promising new therapeutic approach for the treatment of therapy resistant rhabdoid tumors.

## Abbreviations

AT/RT: Atypical teratoidrhabdoid tumors; CDK: Cyclindependent kinase; CDKi: Cyclin dependent kinase inhibitor; FDA: Food and Drug Administration; FDR: False discovery rate; HDAC: Histone deacetylase; HDACi: Histone deacetylase inhibitor; 4-HPR: 4-hydroy(phenyl)retinamide; MTT- 3: (4,5-Dimethylthiazol-2yl)-2,5-diphenyltetrazoliumbromid; NES: Negative enrichment score; SAHA: Suberoylanilindehydroxamic acid; Tam: Tamoxifen.

## Competing interests

The authors declare that they have no competing interests.

## Authors’ contributions

KK, RU, CB, NM, MH, MJ conducted experiments; KK, HJ, MM, MF designed experiments; DR and MK analyzed expression data; DG and ME set up statistical analyses; KK, HJ, MM, MF wrote the manuscript. All authors read and approved the final manuscript.

## Pre-publication history

The pre-publication history for this paper can be accessed here:

http://www.biomedcentral.com/1471-2407/13/286/prepub

## Supplementary Material

Additional file 1: Figure S1 HDACs are highly expressed on RNA level in primary rhabdoid tumors (n = 23) in comparison to differentiated brain tissue (n = 169) using available gene expression profiles of AT/RT [24] and different normal brain tissues [26]. In addition to Figure 1 HDAC 5, HDAC 6 and SIRT1 are significantly upregulated in rhabdoid tumors compared to normal brain tissue. Click here for file

Additional file 2: Figure S2 A. Flow cytometry analysis: After 18 h treatment, SAHA (10 μM) induces G_2_ arrest and the formation of multinuclear cells (dashed line) after 18 h treatment in G401. After 72 h this G_2_ arrest is reversed (dotted line). B. SAHA (10 μM) treatment results in induction of apoptosis in G401 cells after 72 h. Click here for file

Additional file 3 **To confirm microarray data G401 cells were treated with SAHA (10 μM) for 12 h.** QPCR shows upregulation of “Rb-pathway” associated genes. Click here for file
